# Genomic Surveillance of Ceftriaxone-Resistant *Escherichia coli* in Western New York Suggests the Extended-Spectrum β-Lactamase *bla*_CTX-M-27_ Is Emerging on Distinct Plasmids in ST38

**DOI:** 10.3389/fmicb.2020.01747

**Published:** 2020-07-30

**Authors:** Heba H. Mostafa, Andrew Cameron, Samantha M. Taffner, Jun Wang, Adel Malek, Ghinwa Dumyati, Dwight J. Hardy, Nicole D. Pecora

**Affiliations:** ^1^ Department of Pathology and Laboratory Medicine, University of Rochester Medical Center, Rochester, NY, United States; ^2^ Department of Pathology, Johns Hopkins School of Medicine, Baltimore, MD, United States; ^3^ Department of Medicine, Infectious Diseases, University of Rochester Medical Center, Rochester, NY, United States; ^4^ Department of Microbiology and Immunology, University of Rochester Medical Center, Rochester, NY, United States

**Keywords:** *Escherichia coli*, ceftriaxone, plasmids, bacterial genomics, mobile elements, extended-spectrum beta lactamases, nitrofurantoin

## Abstract

Extended-spectrum β-lactamase (ESBL)-producing *Enterobacteriaceae* pose significant treatment and infection prevention challenges. *Escherichia coli* sequence type (ST) 131 associated with the *bla*_CTX-M-15_ gene has been the dominant lineage of ESBL-producing *E. coli* in the US and worldwide. In this study, our objective was to determine the β-lactamase profile, means of dissemination, prevalence, and the clonal identity of ESBL-producing *E. coli* in our region of Western New York. Whole-genome SNP-based phylogenomics was used to assess 89 ceftriaxone-resistant (CTR) *E. coli*. Isolates were collected from both inpatients and outpatients and from urine and sterile-sites over a 2 month period in 2017 or throughout the year, respectively. ST131 was the predominant ST (46.0%), followed by ST38 (15.7%). The *bla*_CTX-M-15_ gene was commonly found in 53.7% of ST131 isolates, whereas the *bla*_CTX-M-27_ gene was found in 26.8% of ST131, though was significantly associated with ST38, and was found in 71.4% of those strains. When compared to ST131, ST38 *E. coli* exhibited increased frequency of resistance to nitrofurantoin and decreased frequency of resistance to ciprofloxacin and ampicillin-sulbactam. Using Nanopore long-read sequencing technology, an analysis of the ESBL genetic context showed that the *bla*_CTX-M-15_ gene was chromosomal in 68.2% of ST131, whereas the *bla*_CTX-M-27_ gene was plasmid-borne in all ST131 and 90% of ST38 isolates. Notably, the *bla*_CTX-M-27_ gene in ST38 resided on highly-related (99.0–100.0% identity and 65.0–98.0% query coverage) conjugative IncF plasmids of distinct plasmid multi-locus sequence types (pMLSTs) from those in ST131. Furthermore, ST131 and ST38 were found to harbor different antibiotic resistance gene and virulence factor profiles. These findings raise the possibility of an emerging ESBL-producing *E. coli* lineage in our region.

## Introduction

Extended-spectrum β-lactamase (ESBL)-producing organisms are responsible for ~26,000 drug-resistant infections and ~1,700 deaths per year in the US, where they are categorized as a serious and increasing threat within the Centers for Disease Control and Prevention’s (CDC) 2019 Antibiotic Resistance Threat Report (CDC, 2019). Among hospitalized patients, ESBL-producers may account for up to 11.6 and 16.1% of *Escherichia coli* causing urinary tract infection (UTI) and bloodstream infections (BSIs), respectively (Mendes et al., 2019). At present, CTX-M β-lactamases are the prevailing family of ESBLs and include more than 150 genes (Zhao and Hu, 2013). They may have originated as chromosomally-encoded enzymes in *Kluyvera* spp. before spreading to *Escherichia*, *Klebsiella*, and other enteric bacteria (Rossolini et al., 2008). Documented mechanisms of mobilization include capture by the insertion elements IS*Ecp1* and IS*CR1*, as well as bacteriophages (Poirel et al., 2008). While chromosomal integration is reported, CTX-M β-lactamase genes are more frequently associated with IncF plasmids (Doumith et al., 2012; Stoesser et al., 2016).

The most frequently reported gene, *bla*_CTX-M-15_, is associated with uropathogenic *E. coli* (UPEC) sequence type 131 (ST131), the predominant lineage of extra-intestinal pathogenic *E. coli* (ExPEC) worldwide (Nicolas-Chanoine et al., 2014; Pitout and DeVinney, 2017; Birgy et al., 2020). The successful emergence of this clone is attributed to the acquisition of antimicrobial resistance (AMR), specifically to fluoroquinolones (Nicolas-Chanoine et al., 2014; Shaik et al., 2017). In the US, the *bla*_CTX-M-15_ gene has also been frequently reported in the context of ST131 (often carried on IncF plasmids; Chandramohan and Revell, 2012; Banerjee et al., 2013; Doi et al., 2013; Chen et al., 2014; Kanamori et al., 2017). However, hinting that the epidemiology may be changing, the 2016 SENTRY Antimicrobial Surveillance Study demonstrated that the *bla*_CTX-M-27_ gene (17.3%) is also significant in *E. coli* UTI and BSI isolates, compared to the *bla*_CTX-M-15_ gene (55.5%; Mendes et al., 2019). Similarly, ST131 carrying the *bla*_CTX-M-27_ gene is also a frequent minority around the globe (Livermore et al., 2007; Cao et al., 2011; Dahbi et al., 2013; Matsumura et al., 2013; Seiffert et al., 2013; Roer et al., 2017; Guiral et al., 2019; Peirano and Pitout, 2019; Birgy et al., 2020). The relative advantages of one CTX-M family gene vs. another are not clear, though the *bla*_CTX-M-27_ gene may confer additional activity against ceftazidime (Bonnet et al., 2003).

The genetic context of *bla*_CTX-M-15_ and other CTX-M family genes in ESBL-producing *E. coli* in the US remains relatively undefined. In this study, we used bacterial whole-genome sequencing (WGS) to investigate the genomic epidemiology of 89 ceftriaxone-resistant (CTR) *E. coli* with respect to clonality, susceptibility, multi-drug resistance (MDR), and β-lactamase profiles. One of our goals was to compare the prevailing clonal types and ARGs between urine and sterile-site isolates from both inpatients and outpatients. Plasmid CTX-M gene context was further examined for all isolates using long-read sequencing. Complete plasmid sequences and chromosomal integration sites were mapped. Thus, this study represents a detailed snapshot of the genomic landscape, including the apparatus of horizontal transmission, of ESBL-producing *E. coli* isolated in our region of Western New York (NY).

## Materials and Methods

### Clinical Laboratory Setting and Isolate Selection

This study was performed under University of Rochester Medical Center (URMC) IRB protocol RSRB00068143. Eighty-nine CTR *E. coli* isolates collected as part of routine clinical care at the URMC Clinical Microbiology laboratory in Rochester, New York were selected for this project. The laboratory provides diagnostic services to a population of ~0.5 million people in Western NY and services several area hospitals, urgent cares, nursing homes, and outpatient practices. To identify potential ESBL-producing organisms, we selected unique patient isolates for WGS based on CTR. Urine CTR *E. coli* were collected at convenience during the months of October and November in 2017 (53 isolates), and CTR *E. coli* derived from sterile-site infections were collected throughout 2017 (36 isolates: 28 BSIs, 4 bone, 2 peritoneal fluid, 1 joint fluid, and 1 drain). In their respective timeframes, this captured 53/67 (79.1%) of unique patient CTR urine isolates and 36/36 (100%) CTR sterile-site isolates. Initial *E. coli* identification was performed with MALDI-TOF (Vitek MS v3.0; bioMérieux Inc., Durham, NC). Antibiotic susceptibility (including ESBL production) was assessed with Vitek 2 (bioMérieux Inc.; AST-GN70 test card). Phenotypic ESBL production assessed by Vitek2 is indicated by “+” (Data S1 – Antibiogram). Cefazolin susceptibility was determined by Kirby-Bauer disk diffusion for isolates from sterile-sites and is indicated with “/KB” in Data S1 – Antibiogram. Kirby-Bauer zone of inhibition diameter is indicated in millimeters. Susceptibility was interpreted with CLSI standard M100 (CLSI, 2019).

### Bacterial Growth Conditions and Genomic DNA Extraction

*E. coli* isolates were archived at −80°C in trypticase soy broth (TSB) with 20% glycerol and maintained at 35°C on blood agar (BD BBL trypticase soy agar with 5% Sheep Blood; BD). Bacterial DNA was extracted with the MagNA Pure Compact System (Roche, Indianapolis, IN). DNA was quantified with the QuantiFluor dsDNA system (Promega, Madison, WI).

### Genomic DNA Sequencing

DNA library preparation was performed according to the manufacturer’s protocol (Nextera XT DNA Library Preparation Kit; Illumina, San Diego, CA), purified using Agencourt AMPure XP beads (Beckman Coulter Inc., Indianapolis, IN), and quality-checked using the Agilent 4,200 TapeStation System (Agilent; Santa Clara, CA). Purified PCR products were normalized using Nextera XT Library Normalization Beads (Illumina). Normalized samples were pooled and quantified using the Qubit ssDNA Assay kit (Invitrogen). Library pools were loaded with 2.45 ng ssDNA and 20 μl PhiX control DNA (20 pM). Paired-end sequencing was performed with MiSeq Reagent v3 600-cycle kits on the MiSeq instrument (Illumina).

### Plasmid Purification and Long-Read Sequencing

Plasmids were sequenced on the MinION platform (Oxford Nanopore Technologies; Cambridge, MA). Briefly, for each of the 89 strains, 100 ml of Luria-Bertani (LB) broth was inoculated and incubated for ~18 h at 37°C, and plasmids were purified using the QIAfilter Plasmid Midi Kit (Qiagen, Germantown, MD). Plasmid DNA was purified (Agencourt AMPure XP beads), and quantified (QuantiFluor dsDNA), and adjusted to 400 ng in a total volume of 7.5 μl molecular biology-grade H_2_O. Sequencing library preparation (Rapid Barcoding Sequencing kit; Oxford Nanopore Technologies) was performed as indicated with the following alterations: Fragmentation Mix RB01-12 volume was reduced to 1.5 μl and was incubated for 20 s at 30°C. Base-calling and de-multiplexing was performed using Albacore v2.3.1 (Oxford Nanopore Technologies) and Illumina-Nanopore hybrid read assemblies were generated by Unicycler (Wick et al., 2017). Plasmids were denoted as “circular” (complete) or “uncircularized” (i.e., incomplete or fragments). BLASTn was used to identify plasmids, plasmid multi-locus sequence typing (pMLST), and virulence and antimicrobial resistance genes (ARGs), which were also assessed with Center for Genomic Epidemiology (CGE) tools (including ResFinder and FimTyper; Thomsen et al., 2016) and with ABRicate (Seemann, 2017). Plasmids were annotated using RAST (Aziz et al., 2008) and aligned with Mauve (Darling et al., 2004). In some instances, genomic sequences recovered from long-read plasmid sequencing were used to create hybrid assemblies with Illumina reads.

### Bioinformatics and Statistical Analyses

A pipeline written in Python, sqlite3, BASH, JavaScript, D3 (Bostock et al., 2011), JQuery, HTML, and Bootstrap was used for genomic DNA read quality control, genomic sequence assembly, mapping, SNP calling, and phylogenomics. Briefly, human DNA reads were removed using Bowtie2 v2.3.5.1 (Langmead and Salzberg, 2012). Trimmomatic v0.39 (Bolger et al., 2014) was used for adaptor removal and read quality control (with altered criteria: SLIDINGWINDOW: 5:20; and MINLEN: 50). Assemblies were built using SPAdes v3.13.0 (Bankevich et al., 2012). Genome assembly quality was assessed by Quast (Gurevich et al., 2013). Genomes were aligned against *E. coli* MG1655 (GenBank: NC_000913.3) using Bowtie2 (Langmead and Salzberg, 2012). BLASTn was used to determine multi-locus sequence typing (MLST; Jolley and Maiden, 2010). ARGs and virulence factors were determined as above. Phage sequences were detected with PHASTER (Arndt et al., 2016). *E. coli* phylogroups were determined with ClermonTyper (Beghain et al., 2018).

A modified CFSAN SNP Pipeline was used for reference-based SNP calling and phylogenetic analysis (Davis et al., 2015). The “call_sites” function uses samtools (Li et al., 2009) and varscan (settings: min-var-freq:0.90; min-coverage:12; min-avg-qual:20; Koboldt et al., 2012) to find high-confidence SNPs between the reference sequence and mapped reads. SNPs that occur inside phages, mobile elements, and transposons were removed using a custom RAST-annotated gff file (Aziz et al., 2008). Maximum likelihood trees were built using a concatenated SNP fasta file for each analyzed genome (settings: call_consensus, minConsFreq: 0.9). Trees were produced with FastTree (Price et al., 2010). Plasmid content was determined with Roary v3.11.2 (Page et al., 2015) and visualized with hierarchical clustering using Morpheus (Broad Institute, Cambridge, MA). Circular alignment plots were produced with Circos (Krzywinski et al., 2009). Linear alignment gene diagrams were produced with EasyFig (Sullivan et al., 2011).

Statistical calculations were performed in Prism 8 (GraphPad Software, San Diego, CA). Multiple *t*-tests (Holm-Sidak method) were used for comparison of AMR frequencies. Otherwise, Fisher’s exact test was used, including for gene content comparisons between ST131 and ST38. Unless otherwise indicated, data shown represent the mean ± SEM.

## Results

### Regional Burden of Ceftriaxone-Resistant and ESBL-Producing *E. coli*

**Figure 1:**
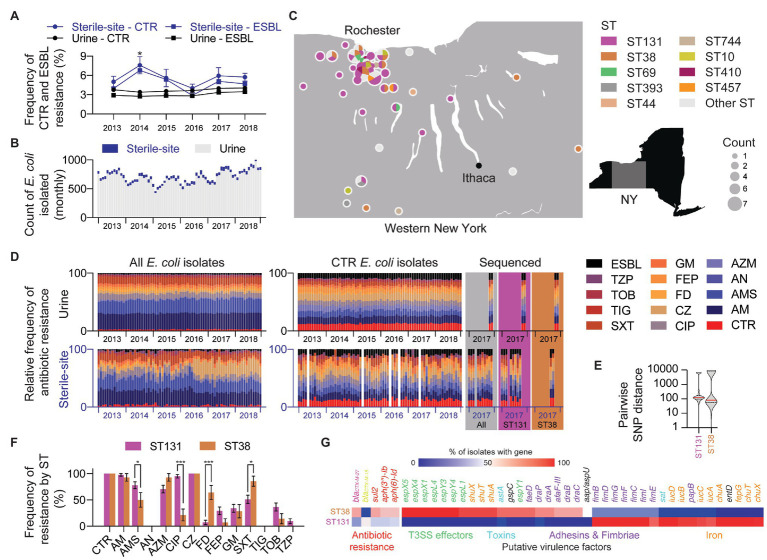
Incidence of ceftriaxone-resistant (CTR) and extended-spectrum β-lactamase (ESBL)-producing *Escherichia coli* in Western New York (NY). **(A)** Yearly frequency of CTR and ESBL-producing *E. coli* in sterile-site and urine *E. coli*. Mean percentage with error bars showing SEM. Statistical test: *t*-test (Holm-Sidak method). Significance: ^*^ *p* ≤ 0.05. **(B)** Total monthly count of sterile-site and urine *E. coli* isolated between 2013 and 2018. Data include only one isolate per patient. **(C)** Geographic distribution and prevalence of *E. coli* sequence types (STs). **(D)** Antimicrobial resistance (AMR) profiles of sterile-site and urine *E. coli* isolated between 2013 and 2018. Relative frequency is shown for monthly profiles for all isolates **(left)**, CTR isolates **(right)**, and whole-genome sequenced (WGS) CTR isolates (colored panels). CTR, ceftriaxone; AM, ampicillin; AMS, ampicillin-sulbactam; AN, amikacin; AZM, aztreonam; CIP, ciprofloxacin; CZ, ceftizoxime; FD, nitrofurantoin; FEP, cefepime; GM, gentamicin; SXT, trimethoprim-sulfamethoxazole; TIG, tigecycline; TOB, tobramycin; TZP, piperacillin-tazobactam; ESBL, Extended spectrum β-lactamase. **(E)** Violin plot showing distribution of intraclade pairwise SNP distances for ST131 and ST38. **(F)** Differences in AMR rates between sterile-site and urine *E. coli* ST131 and ST38 STs. Statistical test: multiple *t*-tests (Holm-Sidak method). Significance: ^*^p ≤ 0.05 and ^***^p ≤ 0.001. **(G)** ARGs and putative virulence factors associated with ST131 and ST38. Color indicates percentage of ST131 or ST38 isolates encoding genes listed. Genes are listed only if mean ST131 and ST38 percentages differ by >30% (for ARGs) and >50% (for putative virulence factors).

Surveying over 5 years (2013–2018), the average frequency (mean ± SEM) of CTR *E. coli* in sterile-site and urine isolates was 5.6 ± 1.2% and 3.7 ± 0.1%, respectively. Likewise, ESBL-producers were detected at frequencies of 4.8 ± 1.3% and 3.0% ± 0.1% for sterile-site and urine isolates (Figure 1A). The majority of CTR *E. coli* were isolated from urine specimens (90.3%; Figure 1B). The sequenced CTR *E. coli* originated across a geographic distribution in Western NY that approximated the patient population served by our laboratory (Figure 1C). Isolates from inpatients accounted for 53.9% of the overall study set. Of sterile-site isolates, 94.4% were from inpatients vs. 26.4% of urine isolates. The cumulative antibiogram for CTR *E. coli* isolated from urine indicated the following resistance frequencies (mean ± SEM): ampicillin (99.9 ± 0.007%), ampicillin-sulbactam (78.4 ± 0.9%), amikacin (0.4 ± 0.1%), aztreonam (69.5 ± 1.0%), ciprofloxacin (65.6 ± 1.0%), nitrofurantoin (13.3 ± 0.8%), cefepime (35.0 ± 1.2%), gentamicin (28.7 ± 1.1%), trimethoprim-sulfamethoxazole (48.6 ± 1.2%), tigecycline (0%), tobramycin (32.2 ± 1.1%), and piperacillin-tazobactam (12.7 ± 0.8%). The resistance frequencies for CTR *E. coli* isolated from sterile-sites were: ampicillin (94.3 ± 3.2%), ampicillin-sulbactam (67.9 ± 6.5%), amikacin (0%), aztreonam (75.5 ± 6.0%), ciprofloxacin (71.7 ± 6.2%), nitrofurantoin (17.0 ± 5.2%), cefepime (22.6 ± 5.8%), gentamicin (20.7 ± 5.6%), trimethoprim-sulfamethoxazole (56.6 ± 6.8%), tigecycline (1.9 ± 1.8%), tobramycin (20.7 ± 5.6%), and piperacillin-tazobactam (37.7 ± 2.6%). Resistance frequencies of the total *E. coli* in our hospital system and community vs. the sequenced isolates are depicted in Figure 1D. For urine isolates, the frequency of ampicillin resistance was different between the larger collection of CTR *E. coli* vs. sequenced *E. coli* (99.8 ± 0.007 vs. 94.3 ± 3.2%, *p* < 0.000001). Otherwise, there were no significant differences observed for any other drug tested when compared to urine isolates selected for sequencing. Likewise, there were no significant differences in the antibiogram between the sequenced sterile-site CTR *E. coli* and the larger collection of sterile-site *E. coli*. Antibiotic resistance profiles of sequenced isolates are summarized in supplemental data (Data S1 – Antibiogram).

### ST131 and ST38 Were the Predominant Sequence Types and Exhibited Differences in Antibiotic Susceptibilities

WGS MLST determined that ST131 was the most frequent (46.0%, 41/89) and ST38 was the second-most frequent (15.7%, 14/89) ST. These were followed by ST69 (4.5%, 4/89) and ST10 (3.4%, 3/89; Data S1 – Genomes). Excluding ST131 and ST38, the remaining sequenced isolates (35/89) belonged to 25 other STs. Diversity of STs was similar between sterile-site and urine isolates with 16 vs. 17 different STs identified, respectively. Among ST131 isolates, 68.3% were isolated from inpatients (including long-term care facilities), whereas 35.7% of ST38 were isolated from inpatients. Although not significantly different, 41.5% of ST131 were sterile-site isolates compared to 21.4% of ST38 (Table 1). All ST131 isolates were phylogroup B2, and all ST38 isolates were phylogroup D.

**Table 1:** Distinguishing features of ST131 and ST38 isolates sequenced in this study.

MLST	ST131 *n* (%)	ST38 *n* (%)	*p* value
Source			
Urine	24 (58.5)	11 (78.6)	Not significant
Sterile-sites	17 (41.5)	3 (21.4)	Not significant
Location			
Inpatient	28 (68.3)	5 (35.7)	Not significant
Outpatient	13 (31.7)	9 (64.3)	Not significant
Phylogroup			
B2	41 (100.0)	0 (0.00)	<0.0001
D	0 (0.00)	14 (100.0)	<0.0001
ESBL			
*bla*_CTX-M-15_	22 (53.7)	1 (7.2)	0.004
*bla*_CTX-M-27_	11 (26.8)	10 (71.4)	0.005
Total isolates	41	14	

SNP-based phylogenetic clustering revealed that the median SNP distance between ST131 and ST38 was 24,641 SNPs ± 3.0 SNPs. The median SNP distance among ST131 isolates was 122 SNPs (range: 0–6,792 SNPs) and 82 SNPs (range: 0–11,470 SNPs) for ST38 isolates (Figure 1E and Data S1 – SNP distance).

Compared to ST131, ST38 isolates from urine demonstrated increased frequency of resistance to nitrofurantoin (70.0 ± 15.3 vs. 4.1 ± 4.1%, *p* < 0.000001) and trimethoprim-sulfamethoxazole (90.0 ± 10.0 vs. 41.6 ± 10.2%, *p* = 0.00003; Figure 1F). For nine ST38 strains with intermediate or resistant nitrofurantoin phenotypes, Q67STOP (eight isolates) and Q26STOP (one isolate) truncations were detected in the nitroreductase gene *nfsA* (data not shown). Likewise, mutations in *nfsA* (L60STOP, Q26STOP, and Q113STOP) were detected in three nitrofurantoin-resistant ST131 isolates (data not shown). Increased frequency of trimethoprim-sulfamethoxazole resistance correlated with the presence of *sul1*, *sul2*, and *dfrA* genes, which, when considered together, were more frequent in ST38 than in ST131 (69.1 vs. 28.7%, *p* < 0.0001; Figure 1G). ST38 isolates from urine were also more often susceptible to ampicillin-sulbactam compared to ST131 (40.0 ± 16.3 vs. 79.1 ± 8.4%, *p* = 0.0006). However, *bla*_TEM_ and *bla*_OXA-1_ genes were also absent in both ST38 and ST131 isolates susceptible to ampicillin-sulbactam (Data S1 – Genomes).

ST131 isolates were more frequently resistant to ciprofloxacin than ST38 strains regardless of urine vs. sterile-site specimen type (urine: 95.8 ± 4.1 vs. 30.0 ± 15.2%, *p* < 0.000001; sterile-site: 94.1 ± 5.8% vs. none detected, *p* = 0.000004). Mutations known to be associated with quinolone resistance were detected in ST131 isolates, including in *gyrA* (D87N, S83L, and A828S), and in the *parC* gene (S80I, E84V; Data S1 – ST131; Sorlozano et al., 2007; Phan et al., 2015; Kanamori et al., 2017; Nicolas-Chanoine et al., 2017). Fluoroquinolone resistance correlated with ST131 clades. URMC_16, URMC_22, and URMC_112 demonstrated full or intermediate susceptibility to fluoroquinolones, harbored single *gyrA* mutations, and were found to be *fimH41*, all consistent with Clade A (Data S1 – ST131). The remaining ST131 isolates were all resistant to ciprofloxacin and were characterized as *fimH30* with one exception (*fimH34*, URMC_111), placing them within ST131 Clade C (Data S1 – ST131). Three ciprofloxacin-resistant ST38 isolates had *gyrA* (S83L) and *parC* (S80I) gene mutations.

### Differences in Gene Content Between ST131 and ST38

Profiling for putative virulence factors revealed differences in gene content between ST131 and ST38 isolates (Figure 1G). The majority of ST131 isolates carried genes encoding the secreted autotransporter toxin (*sat*: ST131, 100% vs. ST38, 7.1%; *p* < 0.0001; Guignot et al., 2007), genes for iron acquisition (e.g., *iucC*: ST131, 92.6% vs. ST38, 7.1%; *p* < 0.0001), and genes for Type 1 fimbriae biogenesis (e.g., *fimI*, *fimC*, *fimD*, *fimF*, and *fimG*: ST131, 100% vs. ST38, 21.4%; *p* < 0.0001). Additionally, cytotoxic necrotizing factor (*cnf1*: ST131, 29.3% vs. ST38, 0%; *p* = 0.02) and the plasmid-encoded enterotoxin (*senB*: ST131, 46.3% vs. ST38, 7.1%; *p* = 0.01; Nataro et al., 1995) were also enriched in ST131 isolates, compared to ST38. In contrast, ST38-enriched genes included determinants characteristic of enteroaggregative *E. coli* (EAEC; Lima et al., 2013; Chattaway et al., 2014; Havt et al., 2017), such as genes for aggregative heat stable toxin (*astA*: ST131, 0% vs. ST38, 92.9%; *p* < 0.0001; Paiva de Sousa and Dubreuil, 2001), non-fimbrial adhesin (*afaF*: ST131, 0% vs. ST38, 71.4%; *p* < 0.0001; Korotkova et al., 2008), iron transport (*shuA*, *shuT*, *shuX*: ST131, 0% vs. ST38, 92.9%; *p* < 0.0001), and anti-aggregation protein (*aap*: ST131, 0% vs. ST38, 50.0%; *p* < 0.0001; Sheikh et al., 2002).

### ST131 and ST38 Have Distinct CTX-M β-Lactamase Genes

**Figure 2:**
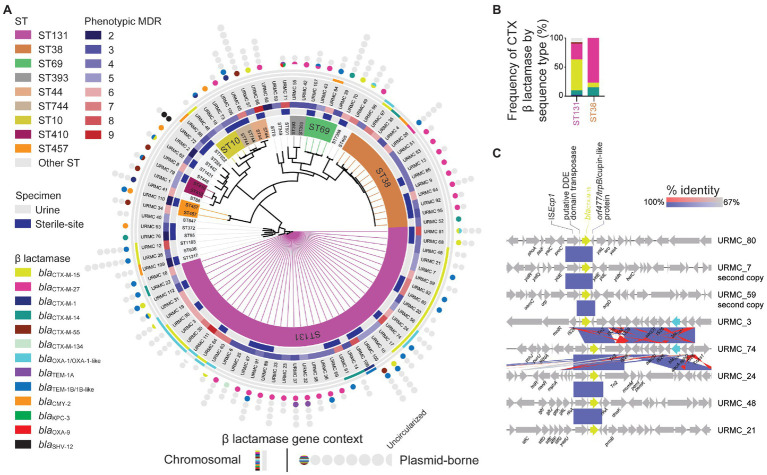
WGS SNP-based phylogenomic tree and ST distribution of sequenced CTR *E. coli*. **(A)** Isolates (89 total) selected for WGS included those from urine (53 isolates) and sterile-sites (36 total: 28 blood, 4 bone, 2 peritoneal fluid, 1 joint fluid, and 1 drain) specimens. Detected ARGs are displayed as chromosomal (lines) or plasmid-borne (circles). Circles indicate the number of plasmids identified in each *E. coli* isolate. Half-circles indicate incomplete sequences. **(B)** Relative frequency of β-lactamase genes detected in *E. coli* ST131 and ST38 STs. **(C)** Gene context schematic of representative *bla*_CTX-M-15_ gene chromosomal insertion sites.

Among the 89 sequenced isolates, the *bla*_CTX-M-15_ gene was found in 30/89 (34%) isolates (ST131, 22/41 vs. ST38, 1/14; *p* = 0.004; Figure 2A and Data S1 – Genomes). The *bla*_CTX-M-27_ gene was nearly as common and was found in 27/89 (30.3%) isolates. Whereas the *bla*_CTX-M-15_ gene was mainly associated with ST131, the *bla*_CTX-M-27_ gene was associated with both ST131 and ST38, accounting, respectively, for 26.8 and 64.3% of those isolates (ST131, 11/41 vs. ST38, 10/14; *p* = 0.005; Figure 2B and Table 1). Less frequent *bla*_CTX-M_ genes included the *bla*_CTX-M-14_ gene (7/89), the *bla*_CTX-M-55_ gene (6/89), and the *bla*_CTX-M-1_ gene (4/89). Non-CTX-M family β-lactamase genes were found in 9/89 isolates, including the *bla*_CMY-2_ gene (8/89), the *bla*_SHV-12_ gene (1/89), and a *bla*_KPC-3_ gene (1/89). Of 21/41 ST131 strains, the *bla*_CTX-M-15_ gene correlated with *fimH30* and ciprofloxacin resistance, placing those strains in clade C2 (*H*30-Rx; Price et al., 2013; Petty et al., 2014; Stoesser et al., 2016). Of the remaining ST131 isolates, 12.2% (5/41) were Clade C1 (*H*30-R), 26.8% (11/41) were clade C1-M27 (*H*30-R), 7.3% (3/41) were clade A, and one isolate (URMC_111, *fimH34*) was of an undefined clade (Data S1 – ST131). Within the most common MLST, ST131, the *bla*_CTX-M-15_ gene was not more likely to be found among inpatients than the *bla*_CTX-M-27_ gene (63.6 vs. 72.7%; *p* = not significant).

### Genetic Context of the ESBL *bla*_CTX-M-15_ in ST131 and ST38 *E. coli*

Among the 30 isolates with the *bla*_CTX-M-15_ gene, 15/22 ST131 isolates had the gene integrated into the chromosome. The *bla*_CTX-M-15_ gene was chromosomal in 4/8 isolates of other STs, one of which was ST38 (Figure 2A and Data S1 – Genomes). For most chromosomal insertions, alignment (with 100% identity) of the immediate *bla*_CTX-M-15_ context revealed the presence of the well-known IS*Ecp1*-flanked *bla*_CTX-M-15_-*orf477* arrangement (Rodríguez et al., 2004). The *bla*_CTX-M-15_ gene insertions were also typically flanked by other IS element insertions and scars (Figure 2C).

Among the ST131 isolates with chromosomal *bla*_CTX-M-15_, the gene was frequently located in one of two sites, either: (1) between genes encoding shikimate kinase (*aro*) and pyrroline-5-carboxylate-reductase (*proC*; 6/15 isolates); or (2) inserted adjacent to a molybdate metabolism regulator gene (*molR*; 4/15 isolates). In the remaining 5/15 ST131 isolates with chromosomal integration, the *bla*_CTX-M-15_ gene was inserted in unique sites. In two instances, long-read hybrid assembly using chromosomal reads obtained during plasmid sequencing also identified one additional chromosomal copy of the *bla*_CTX-M-15_ gene inserted into unique locations (in URMC_7 and URMC_59; Figure 2C).

**Figure 3:**
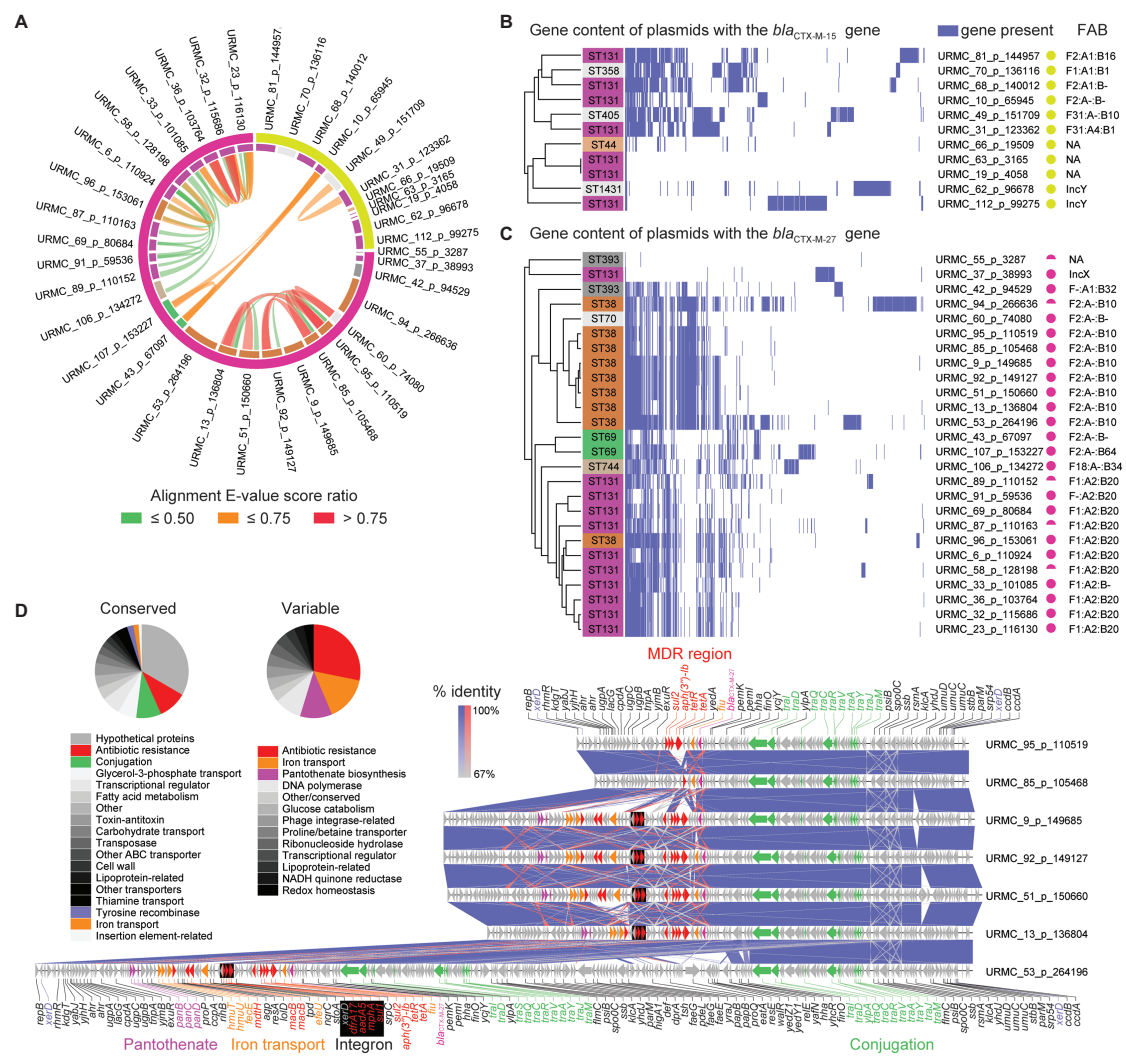
Plasmids harboring the *bla*_CTX-M-15_ and *bla*_CTX-M-27_ β-lactamase genes. **(A)** Circular alignment plot of plasmids with either *bla*_CTX-M-15_ or *bla*_CTX-M-27_ genes showing *E. coli* ST (inner ring; color key consistent with Figure 2A) and β-lactamase (outer ring). Ribbons colored by alignment E-value score ratio. **(B)** Gene content of all plasmids harboring the *bla*_CTX-M-15_ gene. Detected genes shown in blue with hierarchical clustering. **(C)** Gene content of all plasmids harboring the *bla*_CTX-M-27_ gene. Detected genes shown in blue with hierarchical clustering. **(D)** Gene schematic and linear alignment of complete plasmids encoding the *bla*_CTX-M-27_ gene from ST38 (with genes shown as arrows). Arrows are colored to show antibiotic resistance genes (red), conjugation-associated genes (purple), and other/hypothetical genes (gray). Pie charts show relative frequencies of annotated genes by functional characteristics found in conserved **(left)** and variable **(right)** regions in plasmids with the *bla*_CTX-M-27_ gene.

In 6/11 isolates, the plasmid-encoded *bla*_CTX-M-15_ genes were carried on IncF-type plasmids of a variety of pMLSTs (Figure 3B). Two additional plasmids (URMC_62_p_96678 and URMC_112_p_99275) were typed as IncY (Data S1, Plasmids). In general, *bla*_CTX-M-15_ plasmids shared little synteny with each other or with *bla*_CTX-M-27_ plasmids (Figures 3A,B). The plasmids varied in their carriage and arrangement of ARGs. Most (9/11) carried MDR regions with up to 10 ARGs, typically including a Class 1 integron (Data S2). Interestingly, URMC_112_p_99275 carried the *bla*_CTX-M-15_ gene in an intact phage (*Escherichia* phage RCS47; Genbank: NC_042128).

### The *bla*_CTX-M-27_ β-Lactamase Gene Is Associated With Distinct Plasmids in ST38 vs. ST131

The 26 *bla*_CTX-M-27_ β-lactamase genes identified here were borne on plasmids in 11/11 ST131 isolates (clade C1-M27) and 9/10 ST38 isolates (Figure 3C). With the exception of URMC_37_p_38993 (IncX), the 11 ST131 plasmids that encoded the *bla*_CTX-M-27_ gene were of various IncF-types, with pMLST IncF[F1:A2:B20] being found in 9/11 (Figure 3C and Data S1 – Plasmids). Five of these ST131 plasmids had high homology (101,085–116,130 bp, >99% identity) to the multi-replicon plasmid pH105 (134,899 bp; Genbank: CP021871) recently characterized in a vaginal ST131 isolate with the *bla*_CTX-M-27_ gene in Germany (Ghosh et al., 2017). These also carried ARGs acting against aminoglycosides (*aadA5*, streptomycin/spectinomycin resistance; *aph*(6)-Id, tobramycin and amikacin resistance; *aph*(3″)-Ib, streptomycin resistance; and *ant*(3″)-Ia, streptomycin and spectinomycin resistance). Other ARGs detected in the pH105-like plasmids identified here included genes conferring resistance to macrolides [*mph(A)*], tetracyclines [*tet(A)*], sulfonamides (*sul1*, *sul2*), and trimethoprim (*dfrA17*). The MDR regions also encoded a chromate resistance gene (*chrA*), a Class I integron, and were scarred with transpositions of ISs. Typically, the *bla*_CTX-M-27_ gene was flanked by IS*903B* and IS*26*. The other 5 IncF plasmids in ST131 had a variety of close hits in Genbank (Data S1 – Plasmids), with 4/5 bearing no other ARGs other than the *bla*_CTX-M-15_ gene.

The nine ST38 isolates with IncF plasmids carrying the *bla*_CTX-M-27_ gene were highly related (99.0–100.0% identity and 65.0–98.0% query coverage) and ranged in size from 99,138 to 266,636 bp. The majority (8/9) were typed as IncF[F2:A-:B10]. The remaining 1/9 was IncF[F1:A2:B20]. In general, the ST38 plasmids with the *bla*_CTX-M-27_ gene had a conserved ~40-kb conjugation-associated *tra* region and a single complex MDR region (Figure 3D). The MDR region frequently harbored a Class 1 integron stacked with multiple AMR determinants, other ARGs, and genes involved in heavy metal resistance (e.g., *srpC* putative chromate transporter). The ARGs were typically flanked by IS*26* and IS*15D1*. IS associated with the *bla*_CTX-M-27_ gene included IS*903B* and a fragment of IS*Ecp1*, into which IS*26* had inserted. URMC_53_p_264196 was larger (>200-kb) due to a duplication of the *tra* gene region and additional gene content, including a putative enteroaggregative virulence factor (e.g., *astA* heat-stable enterotoxin 1; Figure 3D). In URMC_60_p_74080, the MDR region was not present, and the *bla*_CTX-M-27_ gene was the only ARG on the plasmid.

## Discussion

Our data indicated that the *bla*_CTX-M-15_ gene was the predominant ESBL in our region, but also that the *bla*_CTX-M-27_ gene constituted a large minority, being highly represented in ST131 (26.8%) and in ST38 (64.2%) isolates. Prior studies conducted in the US have indicated that the *bla*_CTX-M-15_ gene is the predominant ESBL, and is frequently carried by ST131, the most widely established extraintestinal clone (Johnson et al., 2012).

In our clade C2 (*H*30-Rx) ST131 isolates, two integration sites accounted for 10/15 chromosomally integrated *bla*_CTX-M-15_ genes. Both of these integration sites have been reported for ESBL *E. coli* collected in other studies (i.e., Genbank: NZ_CP018979 and NZ_CP018991.1). Indicating that these groups did not represent recent local clonal transmission, they were separated by >50 SNPs. The *bla*_CTX-M-15_ gene was carried on a diverse group of plasmids in the clade C2 group, all with unique pMLSTs (Figure 3B).

Others have shown the increasing prevalence of *bla*_CTX-M-27_ in ST131 (Matsumura et al., 2015). In these studies and others, the *bla*_CTX-M-27_ gene has been associated with *fimH30* and fluoroquinolone resistance as part of clade C1-M27. Here, we found the same, as well as noting that the *bla*_CTX-M-27_ gene was often embedded in plasmids of pMLST IncF[F1:A2:B20] which was almost exclusively restricted to the C1-M27 clade, as reported by others (Ghosh et al., 2017; Kondratyeva et al., 2020). Interestingly, An IncF[F1:A2:B20] plasmid was also found in a single ST38 strain (URMC_96; Figure 3C).

Among ST38 strains in our study set, the *bla*_CTX-M-27_ gene was borne on plasmids with few close homologs in Genbank, and which were distinct from those in ST131. These were among the most novel of all the plasmids described in this study. The closest homologue was the IncF plasmid p7_2.1 (Genbank: CP023821), which shared >99% identity over a query coverage range of 33–61% with the ST38 plasmids that carried the *bla*_CTX-M-27_ gene (Data S1 – Plasmids). The p7_2.1 plasmid was identified in a Swedish study of stool isolates but does not harbor the *bla*_CTX-M-27_ gene. Another ST38 *bla*_CTX-M-27_-carrying plasmid (URMC_96_p153061) matched closely (99% identity and 80% query coverage) to plasmid p4_4.1 from the same study (GenBank: CP023827.1), but also had 100% identity (34% query coverage) to pDA33137-178 from a ST44 isolate (Nicoloff et al., 2019). In our study, IncF plasmids in ST38 were commonly (8/9) IncF[F2:A-:B10], where the *bla*_CTX-M-27_ gene was closely associated with IS*903B* and IS*26*. The latter has been reported to help drive the dissemination of some CTX-M β-lactamase genes (e.g., *bla*_CTX-M-14_; Zhao and Hu, 2013). FAB pMLST assessment of ST38 plasmids has not been widely done, though IncF[F2:A-:B10] has been shown in one study to account for 1/12 isolates of a collection of ST38, where IncF[F1:A-:B33] was more commonly observed (4/12; Shaik et al., 2017).

While ST131 is a well-known epidemic lineage, ST38 has historically been found less frequently in surveillance studies, though that may be changing. For example, in the US, 4.8% of *E. coli* causing UTIs and 6.2% of BSI isolates were typed as ST38 in a recent nationwide surveillance study (Mendes et al., 2019). Furthermore, after selecting isolates with elevated MICs for ceftriaxone, aztreonam, ceftazidime, and/or imipenem/meropenem, ST38 comprised 11.6% of UTI and 16.1% of BSI isolates (Mendes et al., 2019). Studies from Europe, the Middle East, and Asia have also found ST38 to be a significant minority among ESBL-producing *E. coli* (Alghoribi et al., 2015; Hertz et al., 2016; Peirano and Pitout, 2019; Sepp et al., 2019). By comparison, our study found that ST38 comprised 15.7% of total CTR isolates (urine: 18.8%; sterile-site: 11.1%). Human clinical ST38 isolates have previously been associated with *bla*_CTX-M-9_, *bla*_CTX-M-14_, *bla*_NDM-1_, and increasingly, with *bla*_OXA-48_ genes (Suzuki et al., 2009; Kim et al., 2011; Mshana et al., 2011; Poirel et al., 2011; van der Bij et al., 2011; Brodrick et al., 2017), but only sporadically with the *bla*_CTX-M-27_ gene (Flament-Simon et al., 2020; Yasir et al., 2020). Other molecular epidemiological studies have not, to our knowledge, detected this association (Day et al., 2019), indicating that carriage of the *bla*_CTX-M-27_ gene by ST38 isolates may be emerging.

Published genomic comparisons of ST131 with ST38 suggest that the latter harbors fewer UPEC-associated virulence genes, though the two have similar *in vitro* adhesion, invasion, and serum resistance phenotypes (Shaik et al., 2017). Elsewhere, ST38 has been described as an ExPEC or a UPEC/EAEC hybrid (Chattaway et al., 2014; Phan et al., 2015; Muenzner et al., 2016). Others have suggested that EAEC attributes may increase the potential of such strains to cause UTIs (Boll et al., 2013). The ST38 isolates identified in this study did not harbor genes encoding for aggregative adherence fimbriae (AAF) nor for AggR, the transcription factor that regulates AAF biogenesis (Boll et al., 2012). While profiling virulence factors *in silico* is limited by the quality and quantity of available databases, the ST38 isolates in this study did harbor some putative virulence factors that may be associated with aggregation and dispersion in EAEC. For example, ST38 harbored *afaF-III* (Afa/Dr. adhesin family; Muenzner et al., 2016). The pathogenicity and clinical pertinence of *E. coli* expressing Afa/Dr. adhesins in UTIs are well established (Servin, 2014).

In this study, ST131 exhibited increased frequency of resistance to fluoroquinolones and ampicillin-sulbactam compared to ST38, which was often non-susceptible to nitrofurantoin. Increased frequency of fluoroquinolone resistance in ST131 vs. ST38 has been previously described (Alghoribi et al., 2015; Gauthier et al., 2018; Guiral et al., 2019) and is a hallmark of clade C strains. Only 3/41 ST131 isolates in this study were susceptible (S or I) to fluoroquinolones, all of which were clade A. To the best of our knowledge, the increased frequency of nitrofurantoin resistance in ST38 has not been reported. Nonsense mutations in the nitroreductase genes *nfsA* and *nfsB* are associated with nitrofurantoin resistance and were found in all nonsusceptible strains of both ST38 and ST131 (Sandegren et al., 2008; Shanmugam et al., 2016).

If ST38 is emerging as a prominent ESBL lineage, then concurrent resistance to nitrofurantoin is concerning because this drug has thus far remained useful for fluoroquinolone-resistant and ESBL-producing organisms (Hertz et al., 2016; Tulara, 2018). Vice-versa, the emergence of this lineage may be influenced by the reduction of fluoroquinolone use. With respect to the observed differences for ampicillin-sulbactam, others have noted that *bla*_CTX-M-15_ genes (or isolates harboring these determinants) are associated with increased frequency of resistance compared to isolates carrying the *bla*_CTX-M-27_ gene (Faheem et al., 2013; Matsumura et al., 2015). This is consistent with our results given the respective preponderance of these enzymes in ST131 vs. ST38. This observation may also be related to the presence of other β-lactamases, as isolates of both ST131 and ST38 were more often resistant to ampicillin-sulbactam if they also harbored broad spectrum β-lactamase genes such as *bla*_TEM_ and *bla*_OXA_.

Limitations of this study include the regional nature and narrow timeframe of isolate collection. Furthermore, while our sequenced *E. coli* may have similar phenotypic susceptibility to both current and past isolates, they may not be representative of the ARGs, STs, and mobile genetic elements found in the community across time. The findings in this study raise several questions. For example, is there a fitness advantage for isolates carrying the *bla*_CTX-M-27_ gene? This gene may confer greater resistance to ceftazidime (Kuroda et al., 2012). What explains the almost exclusive association between ST38 and the *bla*_CTX-M-27_ gene, while ST131 is associated with both the *bla*_CTX-M-27_ and *bla*_CTX-M-15_ genes? Is the spread of *bla*_CTX-M-27_ in our region associated with human carriage from areas of high prevalence or an isolated clonal outbreak? Recent surveillance of food-producing animals in the US showed that cattle and turkey *E. coli* frequently carried the *bla*_CTX-M-27_ gene (Tadesse et al., 2018). *Salmonella* spp. from food-producing animals have also been shown to carry the *bla*_CTX-M-27_ gene (Zhang et al., 2016), though the plasmids in our study bore little resemblance to publically available sequences from *Salmonella* (data not shown). Establishing a link between these observations highlights the need for more extensive and longitudinal “One Health” surveillance studies. In conclusion, although this work may serve as a window through which to view national epidemiological trends, additional surveillance is needed to confirm the emergence of ST38 and its association with the *bla*_CTX-M-27_ gene.

## Data Availability Statement

The datasets presented in this study can be found in online repositories. The names of the repository/repositories and accession number(s) can be found in the article/Supplementary Material.

## Author Contributions

HM and NP designed the study. HM and NP selected clinical isolates. HM isolated genomic DNA. JW performed sequencing. ST performed and managed bioinformatics analyses and pipelines. HM, AM, and AC analyzed sequence data. HM and AC performed statistical analyses. HM, AC, and NP analyzed overall data/results and wrote the first draft of the manuscript. NP provided funding and resources. NP, GD, and DH provided technical expertise. All authors participated in editing and reviewing the manuscript and approved the final manuscript. All authors contributed to the article and approved the submitted version.

### Conflict of Interest

The authors declare that the research was conducted in the absence of any commercial or financial relationships that could be construed as a potential conflict of interest.

## Supplementary Material

The Supplementary Material for this article can be found online at:

https://www.frontiersin.org/articles/10.3389/fmicb.2020.01747/full#supplementary-material.
