# Supplementary material for: Genomic Surveillance of Ceftriaxone-Resistant Escherichia coli in Western New York Suggests the Extended-Spectrum β-Lactamase blaCTX-M-27 Is Emerging on Distinct Plasmids in ST38
Source: Front Microbiol. 2020 Jul 30;11:1747. doi: 10.3389/fmicb.2020.01747 (PMC7406970; doi:10.3389/fmicb.2020.01747)
Supplement: Supplementary file 2 [file Data_Sheet_2.DOCX]

**Data S2**

**
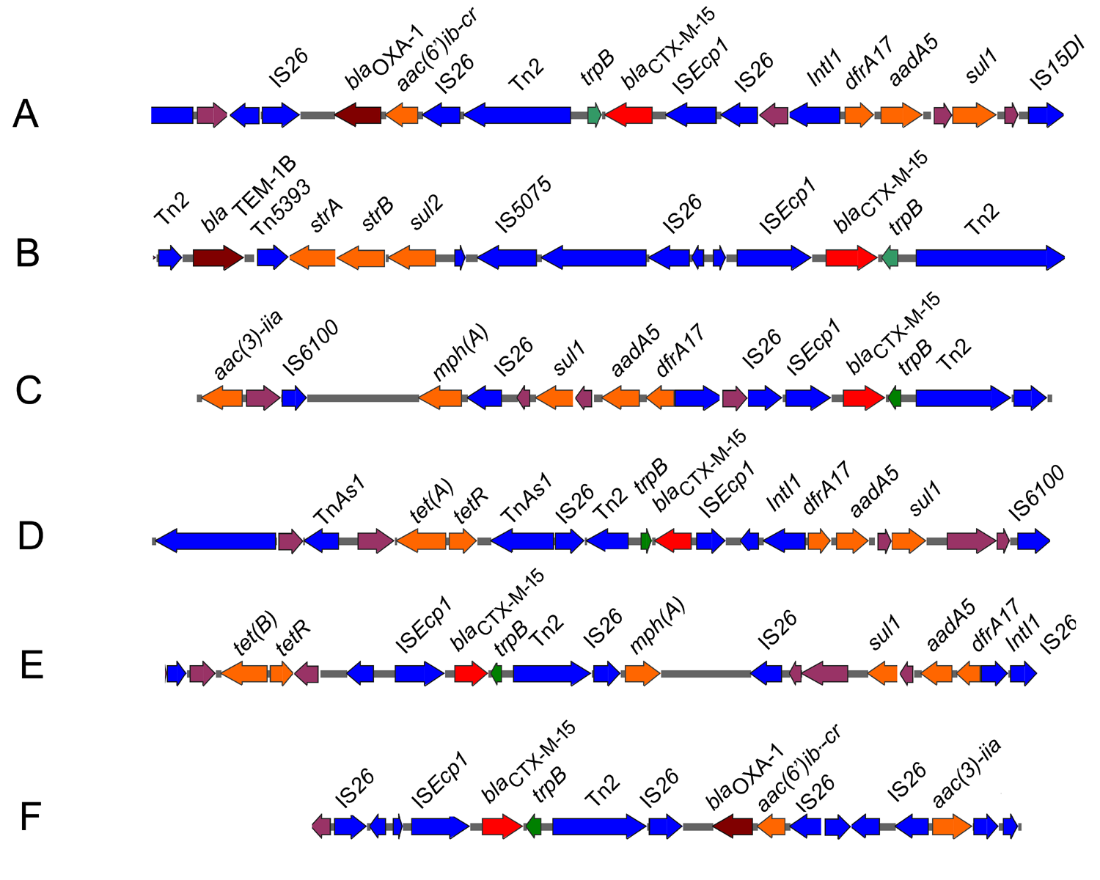
**

***bla*_CTX-M-15_ insertion sites and associated MDR regions in *bla*_CTX-M-15_ carrying plasmids**. *bla*_CTX-M-15_ integration sites were resolved using hybrid assemblies of MinION long reads and Illumina short reads. Regions are shown from plasmids isolated from URMC_49 (A), URMC_62 (B), URMC_66 (C), URMC_68 (D), URMC_70 (E), URMC_81 (F). Insertion sequences are colored in blue, ARGs in orange, and *bla*_CTX-M-15_ in red.


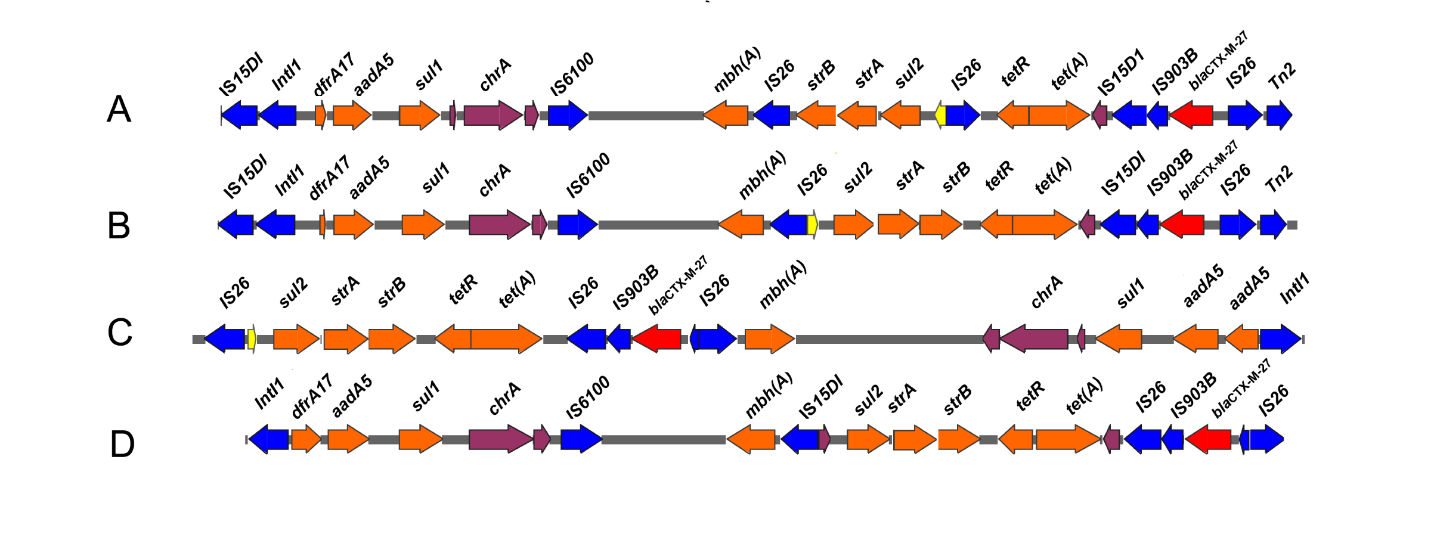


**Sites of *bla*_CTX-M-27_ insertion and associated MDR regions in ST131-*bla*_CTX-M-27_ plasmids**. Hybrid assemblies of MinION long reads and Illumina short reads were used to complete plasmid sequences. Regions are shown from plasmids isolated from URMC_23 (A), URMC_32, 33, 36, and 58 (B), URMC_87 (C), URMC_89 (D). Insertion sequences are colored in blue, ARGs in orange, and *bla*_CTX-M-27_ in red.
